# Oral Microbiota Changes in Elderly Patients, an Indicator of Alzheimer’s Disease

**DOI:** 10.3390/ijerph18084211

**Published:** 2021-04-15

**Authors:** Yi-Fan Wu, Wei-Fang Lee, Eisner Salamanca, Wan-Ling Yao, Jo-Ning Su, Sin-Yu Wang, Chaur-Jong Hu, Wei-Jen Chang

**Affiliations:** 1 School of Dentistry, College of Oral Medicine, Taipei Medical University, Taipei 110, Taiwan; yfwu@tmu.edu.tw (Y.-F.W.); eisnergab@hotmail.com (E.S.); yaoyao061637@gmail.com (W.-L.Y.); m204109002@tmu.edu.tw (J.-N.S.); b202108017@tmu.edu.tw (S.-Y.W.); 2 School of Dental Technology, College of Oral Medicine, Taipei Medical University, Taipei 110, Taiwan; weiwei@tmu.edu.tw; 3 Department of Neurology, School of Medicine, College of Medicine, Taipei Medical University, Taipei 110, Taiwan; 4 Department of Neurology, Shuang Ho Hospital, Taipei Medical University, New Taipei City 23561, Taiwan; 5 Dental Department, Shuang-Ho Hospital, Taipei Medical University, New Taipei City 23561, Taiwan

**Keywords:** oral microbiota, Alzheimer’s disease, plaque/plaque biofilms, oral health, dental hygiene, neuroscience/neurobiology

## Abstract

Alzheimer’s disease (AD) is a neurodegenerative disease that usually affects older individuals. Owing to the higher incidence of root caries and missing teeth in elderly individuals, the bacteria involved in these dental concerns might potentially deteriorate their cognitive function. Altered microbiota in the oral cavity may induce neuroinflammation through migration from the oral cavity to the brain. However, the correlation between the composition of the oral microbiota and neurodegenerative disease remains unclear. In this study, we evaluated sequence to determine the relative abundance and diversity of bacterial taxa in the dental plaque of elderly patients with AD and controls. Oral samples; the DMFT index; and other clinical examination data were collected from 17 patients with AD and 18 normal elderly individuals as the control group. Patients with AD had significantly more missing teeth and higher dental plaque weight but lower microbial diversity than controls. Significantly increased numbers of *Lactobacillales*, *Streptococcaceae*, and *Firmicutes*/*Bacteroidetes* and a significantly decreased number of *Fusobacterium* were observed in patients with AD. In conclusion, using the PacBio single-molecule real-time (SMRT) sequencing platform to survey the microbiota dysbiosis biomarkers in the oral cavity of elderly individuals could serve as a tool to identify patients with AD.

## 1. Introduction

Alzheimer’s disease (AD) is a type of dementia that usually occurs in elderly individuals [1]. The symptoms of AD include cognitive decline, especially evident in memory deficit, communication problems, impaired performance of daily life activities, and behavioral and psychological symptoms. The main cause of AD is accumulation of the insoluble fragments of amyloid β in the extracellular plaques and tau protein in the intracellular neurofibrillary tangles in the brain [2]. Several risk factors, including aging, sedentary lifestyle, nutritional deficiency, and genetic variation, have been reported to contribute to the pathogenesis of AD [3]. Advances in technology have enabled identification of the early stage of the disease through invasive or radioactive examinations such as cerebrospinal fluid examination, which entails lumbar puncture and amyloid positron emission tomography.

Although compelling evidence has demonstrated that having a relatively high number of missing teeth is associated with a higher risk of both dementia and mild cognitive impairment among elderly individuals [4], the actual cause of this association is unclear. The imbalance of microbiota composition in the gut could lead to dysbiosis in the host and induce the development of AD through the gut–brain axis [5]. There are three types of dysbiosis which often occur simultaneously including (1) loss of beneficial organisms, (2) excessive growth of potentially pathogenic bacteria, and (3) reduction in total microbial diversity [6]. Proinflammatory cytokines and interleukins are secreted and promote neuroinflammation, facilitating the invasion of the brain by potential pathogens through blood circulation [7]. However, the microbiota composition in patients with AD has not been fully investigated and characterized. A few recent studies have shown that oral microbiota may be associated with AD owing to the shorter route to the brain than that through the gut. For example, Dominy et al. [8] discovered that *Porphyromonas gingivalis,* which is a keystone pathogen of periodontitis in the brain of patients with AD, produces neurotoxic, proteolytic enzymes of the gingipain family and passes through the blood–brain barrier (BBB) from gingival ulceration. Consequently, tumor necrosis factor-α as well as a proinflammatory cytokines (interleukin IL-1β, IL-6, or IL-8) are secreted by potential bacteria such as *Porphyromonas gingivalis* or *Streptococcus mutans*, eventually causing neuroinflammation and neurodegeneration via the bloodstream [9]. Therefore, poor oral health with unbalanced oral microbiota has a major effect on neuroinflammation and induces further neurodegeneration [10,11].

Oral microbiota research has attracted attention over the last decade. In the past, microbial communities were examined after DNA extraction and polymerase chain reaction (PCR) amplification for periodontal diseases and in cases of oral squamous cell carcinoma [12,13]. Next-generation-sequencing (NGS) platforms such as Illumina’s Solexa, Hiseq Miseq, or the Roche 454 sequencing system [14], provide different regions of 16S rDNA sequencing by generating millions of reads. Studies have used saliva [15,16], subgingival plaque [17], and supragingival [18,19] plaque and investigated in the specific target region (i.e., V1–V2, or V3–V4) rather than the full-length region (V1–V9). However, a limitation of the NGS platform is that it generates sequences measuring approximately 100 bp to 500 bp in length for reads rather than 1–3-kb-long base pairs, leading to low-quality data.

Third-generation sequencing technology with the PacBio sequencing platform (PacBio SMRT; Pacific Biosciences of California, Inc., Menlo Park, CA, USA) was recently designed for accurate analysis of the composition of microbial communities [20]. This technique uses a single-molecule real-time (SMRT) method for sequencing and is able to generate long reads over 10,000 bp. Another advantage of this method is that it enables resolution of operational taxonomic units (OTUs) and helps uncover pathogenic variants from the V1–V9 hypervariable regions. This technique has an average error rate lower than 1% after annealing with the SMRTbell adapter, and the single-molecule consensus sequence is then generated. A disadvantage of this technology is the high cost compared with the traditional NGS technologies. Thus far, few studies have investigated the oral microbial diversity and community in the elderly population by using the PacBio SMRT platform.

Although some of studies have investigated the possible link between poor oral hygiene and AD, whether potential pathogens such as *Streptococcus mutans* [21,22] and *P. gingivalis* [8,23] are plausible risk factors for amyloidosis remains controversial. In this study, we used third-generation sequencing technology to characterize the oral microbiota from dental plaque samples of patients with AD and normal elderly individuals as the controls. The oral microbiota was characterized to identify the association between specific microbiota composition and AD.

## 2. Materials and Methods

### 2.1. Sample Collection

Dental plaque samples were collected from 17 patients with AD and 18 controls without AD. The plaque was collected by a trained dentist with periodontal curettes (Gracey curette. Hu-Friedy, USA) from the tooth’s supra-gingival surfaces without activating it on the tooth’s buccal and lingual/palatal at all teeth [24,25]. All participants were all middle-class native Taiwanese. AD was diagnosed by neurologists based on the National Institute on Aging and Alzheimer’s Association criteria [26]. Elderly individuals who visited dental clinics for dental health check-ups and had no cognitive complaints were considered controls. The major exclusion criteria in this study included confounding neurological (e.g., Parkinson syndrome) diseases, oral cancer diseases (e.g., OSCC, osteosarcoma, and salivary gland cancer), and antibiotic administration within the previous two months. AD participants underwent an oral health status check and were assigned scores based on their cognitive and functional activity, which was assessed through tests such as the Clinical Dementia Rating (CDR) scale, Sum of Boxes (SOB), and Mini–Mental State Examination (MMSE). The number of teeth and the Decayed, Missing, and Filled Teeth (DMFT) index were also assessed. The DMFT index represents the caries experience and status of an individual. Additionally, dental plaque weight was measured by digital electronic microbalance. The study protocol was approved by the Taipei Medical University Joint Institutional Review Board (Approval No. N201802070).

### 2.2. Metagenomic DNA Extraction 

Genomic DNA was extracted using a GenElute Bacterial Genomic DNA Kit (Sigma-Aldrich, St. Louis, MO, USA). Each dental plaque sample was treated according to the manufacturer’s protocol of bacterial preparation. The final samples were added to collection tubes with elution solution (10 mM Tris-HCl, 0.5 mM EDTA, pH 9.0) for 16S amplicon sequencing. Next, the extracted DNA concentrations were measured using Nanodrop 2000 (Thermo Fisher Scientific, Waltham, MA, USA) and stored at −20 °C until the next analysis.

### 2.3. Library Construction and Sequencing 

The diversity of the bacterial communities associated with dental plaque was analyzed using SMRT PacBio sequencing technology (Pacific Biosciences, Menlo Park, CA, USA). The full-length 16S rDNA sequence was amplified using the bacterial-specific universal PCR primer 27F and 1492R, following the manufacturer’s instructions. The final products were purified using AMPure PB beads (Agencourt, Beverly, MA, USA; Part Number PB100-265-900) and assessed using a Qubit 2.0 Fluorometer and a Qubit dsDNA HA Assay Kit (Thermo Fisher Scientific).

### 2.4. PacBio Sequencing Data Analysis

The raw reads were processed using pbccs (v.3.4.0) in the SMRT Link Analysis v6.0.0, which generated consensus sequences with low error rates. The sequence data were processed using the QIIME version 1.9.0. OTUs were picked using mothur v.1.39.5, with 97% identity. Chimera sequences were identified using UCHIME v4.2 with the Gold database and by creating an OTU table [27]. The microbial analyses of our samples were based on the Human Oral Microbiome Database (www.HOMD.org accessed on 23 July 2020) and 16S RefSeq Version 15.2 database [28]. To avoid biases generated by unequal depth of sampling, the OTU table was rarefied to an even depth of approximately 10,000 sequences per sample. After the sequencing data were rarefied, four different metrics were calculated for alpha diversity: “Observed species” estimated the amount of OTUs found in each sample, “Chao1” estimated the richness of the species, “Shannon index” calculated the entropy, and “Unweighted Pair Group Method with Arithmetic Mean,” which is a simple agglomerative from the bottom-up hierarchical clustering method, was used for classification of samples based on their pairwise similarity.

Linear discriminant analysis effect size (LEfSe) was used as a tool to find biomarkers of accessible bacteria between patients with AD and controls by using relative abundance [29]. The effect size was estimated through linear discriminant analysis (LDA) in different groups, and each LDA bar length was represented by a log_10_ transformed LDA score. LEfSe identified discriminative features (LDA score ≥2) with significantly varying relative abundance.

## 3. Results

### 3.1. Participants and Their Oral Health Status

A total of 35 participants (17 patients with AD and 18 elderly controls) were recruited in this study. Table 1 shows the background and dental information of the participants in each group. The average age of the patients with AD was 77.9 years and that of the controls was 65.2 years, with no statistically significant difference. In the AD group, the average CDR and MMSE scores in the cognitive assessment were 1 and 14, respectively. In addition, the number of missing teeth and dental plaque weight were significantly higher in the AD group than in the control group. However, no statistically significant difference in the DMFT index was identified between the groups.

### 3.2. Decreased Oral Microbial Diversity in AD Group

**Figure 1:**
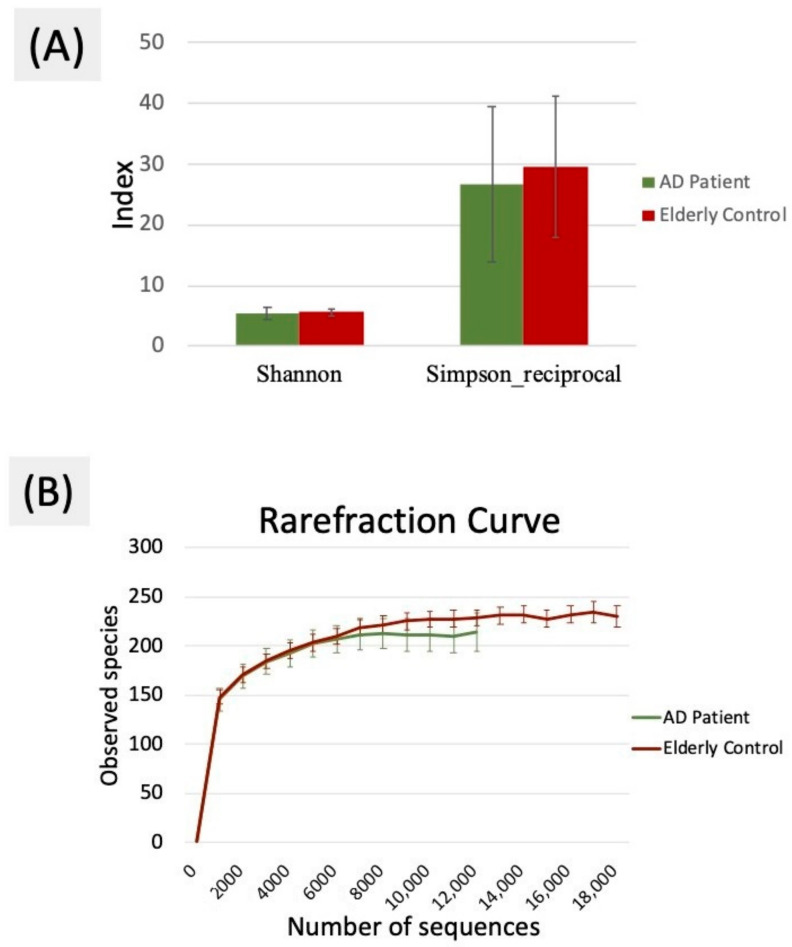
(**A**) Shannon and Simpson indices were used to estimate the level of diversity of the oral microbiota (data shown as the mean ± SD). (**B**) Alpha rarefaction curves were used to estimate the observed species of the oral microbiota of the AD and control groups.

Approximately 10,000 valid unique tags remained in the two groups after quality trimming and filtering (Table 2). The Good’s coverage value was above 99.6% in these two groups, indicating that the sequencing depth was sufficient to observe the oral bacterial communities in patients with AD. The unique tags and number of OTUs were significantly lower in the AD group than in the control group (*p* < 0.05). In addition, the indicators of alpha diversity, including Simpson reciprocal and Shannon indices, representing the level of diversity also showed that the oral microbiota diversity in the AD group was less than that in the controls, as shown in Figure 1A. The alpha rarefaction curve was plotted as the number of distinct OTUs versus the number of sequences to estimate the species richness of the oral microbiota, as shown in Figure 1B. The representation in Figure 1B agrees with the indicators of diversity from Table 2, demonstrating that the overall oral microbial diversity in the AD group tended to be lower than that in the control group.

### 3.3. Taxonomic Classification of OTUs at Phylum and Order Level

**Figure 2:**
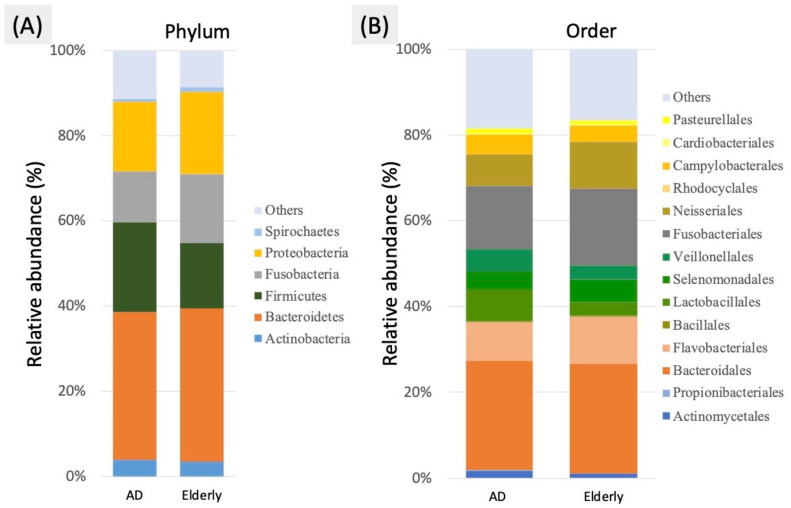
Average bacterial profile of OTUs of oral dental plaque in oral microbiota from the AD and control groups shown by the (**A**) phylum level bar plot (**B**) order level bar plot.

**Figure 3:**
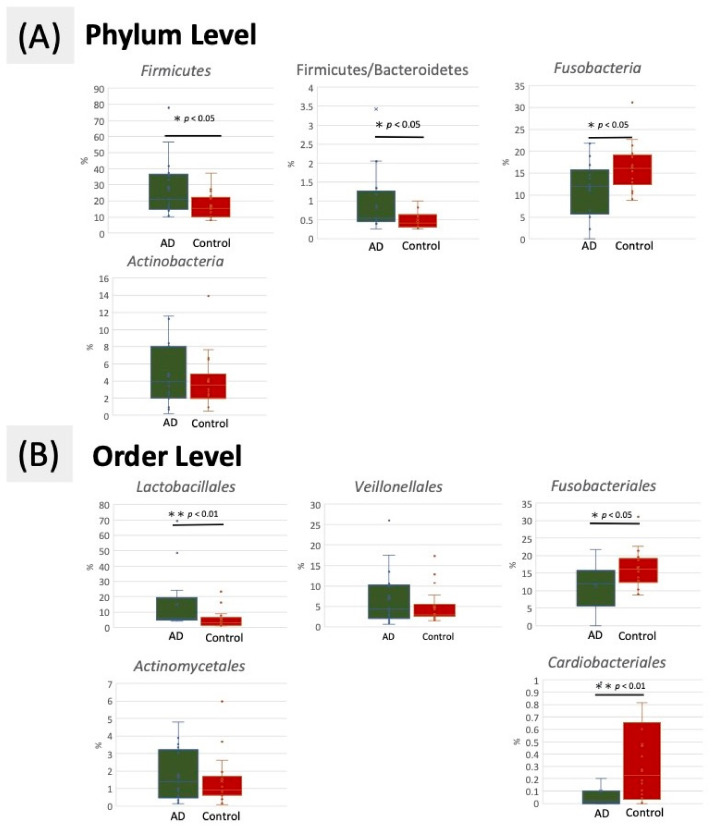
Comparison of relative abundance at the oral bacterial (**A**) phylum and (**B**) order levels in the AD and control groups. * *p* < 0.05 and ** *p* < 0.01 respectively, using Mann–Whitney U test.

The predominant taxa from the dental plaque samples of oral microbiota in the AD and control groups at the phylum and order levels are shown in Figure 2. At the phylum level, the proportion of *Firmicutes* and the ratio of *Firmicutes* and *Bacteroidetes* (F/B ratio) were significantly increased in the AD group compared with the control group, but the proportions of *Fusobacteria* and *Bacteroidetes* were decreased (Figure 3A). An F/B ratio greater than 1 represents systemic inflammation [30]. At the order level, the proportion of *Lactobacillales* was significantly increased in the AD group compared with the control group. A significantly enriched relative abundance of *Actinomycetales* and *Veillonellales* was observed in the AD group. By contrast, *Fusobacteriales* and *Cardiobacteriales* were significantly decreased in the AD group compared with the control group (Figure 3B).

### 3.4. Taxonomic Classification of OTUs at Family and Genus Level

**Figure 4:**
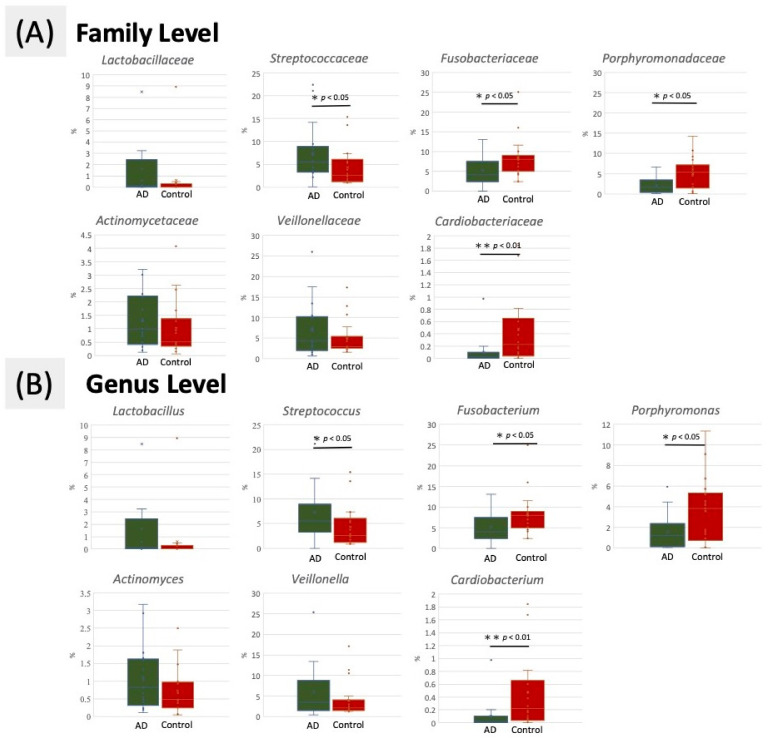
Comparison of relative abundance at the oral bacterial (**A**) family and (**B**) genus levels in the AD and control groups. * *p* < 0.05 and ** *p* < 0.01 respectively, using Mann–Whitney U test.

At the family level, members of *Lactobacillaceae*, *Streptococcaceae*, *Actinomycetaceae*, and *Veillonellaceae* were prevalent in the AD group, whereas enriched populations of *Fusobacteriaceae*, *Cardiobacteriaceae*, and *Porphyromonadaceae* were observed in the control group (Figure 4A). At the genus level, the representation of many genera differed in the AD group from the control group, with *Fusobacterium, Cardiobacterium*, and *Porphyromonas* decreased and *Lactobacillus*, *Streptococcaceae*, *Actinomycetaceae,* and *Veillonella* enriched in the AD group compared with control group (Figure 4B).

### 3.5. Differentially Abundant OTUs in LEfSe Algorithm Analysis

**Figure 5:**
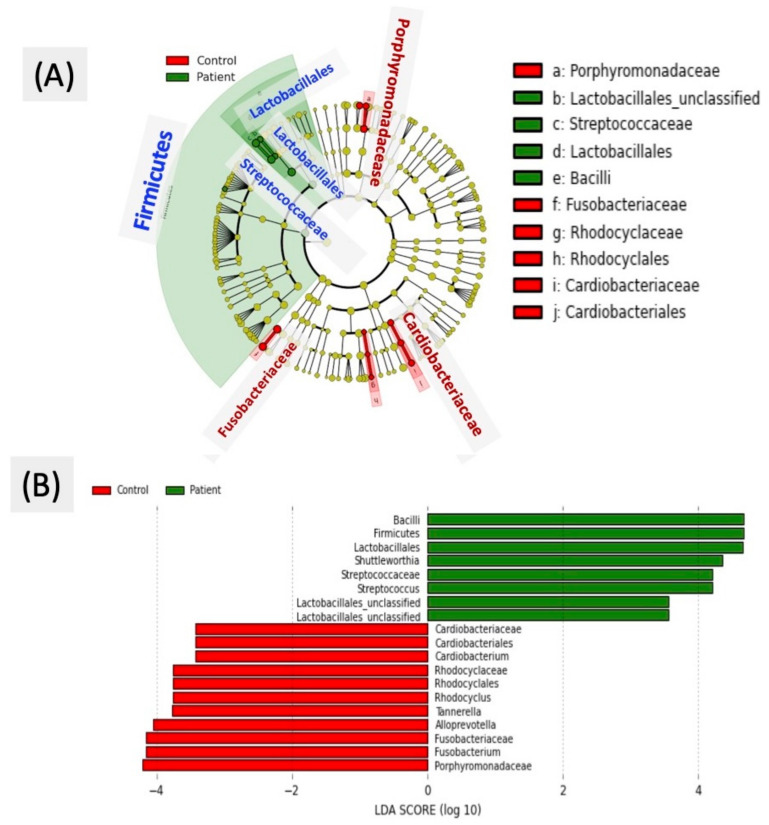
Linear discriminant analysis effect size (LEfSe) was used to identify the most differentially abundant taxa in the AD and control groups (**A**) Taxonomic cladogram of the 16S sequences. The brightness of each dot is proportional to its effect size. (**B**) The taxa that were abundantly found in the elderly control group are indicated with a negative linear discriminant analysis (LDA) score (red), and AD group with a positive score (green). Only the taxa that met a significant LDA threshold value of > |± 3| are shown.

To identify the specific bacterial taxa of oral microbiota related to the AD group, we compared the microbial composition of the two groups by using the LEfSe method. A cladogram representative of the oral microbiota structure and the predominant bacteria is shown in Figure 5A, where the largest differences in the taxa represented in the two communities are displayed. Members of bacterial taxa belonging to *Bacilli*, *Firmicutes*, *Lactobacillales*, *Shuttleworthia*, *Streptococcaceae*, and *Streptococcus* increased in the AD group, and those of *Porphyromonadaceae*, *Fusobacterium*, *Alloprevotella*, and *Cardiobacterium* were enriched in the control group, and thus, these could be used as biomarkers for discrimination between the groups. The LDA bar graph (log 10) for discrimination is presented in Figure 5B.

### 3.6. Correlation between Oral Health and Oral Microbiota in AD

Although neurodegeneration and neuroinflammation are known to influence the imbalance in microbiota changes, the association between oral health, microbiota remains ambiguous in AD. In an attempt to resolve this issue, we found that the changes in the CDR and in the decayed index (*R_s_* = 0.605, *p* < 0.05), and CDR and dental plaque weight correlated positively (*R_s_* = 0.539, *p* < 0.05). In addition, changes in the CDR–Sum of Boxes (CDR-SB) was moderate correlated to the changes in the DMFT index (*R_s_* = 0.538, *p* < 0.05). There was a strong correlation between changes in the DMFT index and in the proportion of *Firmicutes* (*R_s_* = 0.679, *p* < 0.05). Overall, there is a moderate positive correlation between the bad oral condition and cognitive deficits.

## 4. Discussion

Through this investigation, we aimed to evaluate the full-length of a 16S rDNA PacBio SMRT sequence to determine the relative abundance of bacterial taxa from the dental plaque of elderly patients with AD. This platform used in this study generated reads longer than 10 kb and could detect superior taxa at different taxonomic levels. It can provide more reliable results regarding the oral microbial community than the previous short-read-length studies could [31] and a quick method to differentiate the oral bacterial composition between patients with AD and controls; although, it is not as cost-effective as other tests [12]. The oral microbiota is the second most diverse community after the gut microbiota in the human body [32]. In the present study, lower diversity of the oral microbiota indicated dysbiosis in the oral cavity among patients with AD. Previous studies have reported that dysbiosis of the oral microbiota also changes the gut microbiota of the host in patients with obesity [33] and liver diseases [34,35]. The proportion of cariogenic bacteria *Lactobacillale* and *Streptococcaceae* increased in patients with AD (Figure 3B). These pathogenic bacteria in the oral cavity cover a shorter distance to reach the brain than the gut bacteria in the colon do; the shorter distance might interfere with their invasion of the brain [36]. Pathogens from chronically infected sites near the brain are able to penetrate the BBB and easily invade the brain, contributing to neuroinflammation. However, the exact mechanisms of this route of infection are still unclear at present.

The missing teeth count was significantly higher in patients with AD. A high root caries rate and missing teeth are typically correlated with an increased number of oral bacteria [37,38]. As per microbial infection theory, some bacteria can cause tissue inflammation, leading to neuronal death [39]. Another theory has proposed that masticatory reduction can increase the risk of dementia [40]. One study also showed that root caries were associated with tooth loss and influenced the general health of elderly individuals; hence, it is important that dentists take care of these issues [41]. Documenting the natural history of individual teeth and when and why they were lost would facilitate the understanding of the correlation between tooth loss and the development of AD. One study revealed an approximately 80% incidence of root surface caries among adults aged over 65 years [42]. We also found similar results with a moderate correlation between tooth loss and the presence of cariogenic bacteria *Firmicutes*. The phylum *Firmicutes* might be used as a potential diagnostic biomarker based on the results of other studies that also surveyed gut microbiota and found the same relationship between AD and *Firmicutes* in feces [22]. These studies pointed out the importance of determining the mechanism of the effect of microbiota on the pathogenesis of AD. Further, owing to their significantly greater incidence of teeth loss, patients with AD might have a higher mortality rate than that of other elderly individuals [43]. Moreover, a higher F/B ratio (>1) implicated a higher relative proportion of *Firmicutes* than *Bacteroidetes*, which may also reflect the imbalance of the oral cavity microbiota in AD. A higher F/B ratio indicates increased systemic inflammatory response [44] due to the secretion of proinflammatory cytokines, further stimulating neuroinflammation. Therefore, the F/B ratio may also be a good indicator for the early detection of AD, as it is not influenced by the physiological differences among individuals [45]. A study by Ling et al., in 2015 that defined the healthy controls of oral microbial communities from supragingival plaque showed that a major proportion of bacterial sequences from unrelated healthy adults aged between 37 and 59 years were identical, with microbes consisting of the phyla *Firmicutes* (15%), *Actinobacteria* (10%), *Bacteroidetes* (30%), and *Fusobacteria* (27%) [35]. In the present study, controls and patients with AD respectively had 17.3% and 21% *Firmicutes*, 20% and 16.3% *Actinobacteria*, 36.30% and 34.78% *Bacteroidetes*, and 13.73% and 11.98% *Fusobacteria* (Figure 3). Our findings indicated a general increase in *Firmicutes*, *Actinobacteria*, and *Bacteroidetes* and a reduction in *Fusobacteria* when compared with the findings of Ling et al., regarding the oral microbial communities of healthy controls. These variations may be correlated with the changes in oral microbiota that occur with age and maybe considered an indicator of early-onset AD risk, as part of participates included in the study had poor oral hygiene but without any cognitive disease may affect their oral microbiota. The increase in *Firmicutes*, F/B ratio and *Actinobacteria*, but decrease in *Fusobacterium* from healthy adults to elderly patients may indicate that the part of elderly controls recruited in our study already had a cognitive impairment without being officially diagnosed as having AD. Although the cognition was not evaluated in elderly controls, none of them presented signs of cognitive impairment. It might result in insufficient information about the possible severity of cognitive defects but not the diagnosis of AD. However, this part should be documented in further study.

The loss of equilibrium of oral microbiota may occur before the onset of AD. These pathogens might penetrate the BBB, continuously causing neuronal infection and promoting neurodegeneration. The neuroinflammation induced by these pathogens might also cause cognitive impairment and result in the pathogenesis of AD. *Lactobacillus*, *Streptococcaceae*, and *Shuttleworthia* are known to be notable cariogenic dental pathogens that cause root caries and ultimately lead to tooth loss. Postmortem brain examination of patients with AD also showed a significantly increased population of *Actinobacteria*, *Lactobacillus* and *Streptococcaceae* in patients with AD [46]. Controls and patients with AD had a similar proportion of *Actinobacteria* (3.05% and 3.90%, respectively). A similar pilot study using high-throughput DNA sequencing of the V3–V4 region determined the relative abundance of bacterial taxa in subgingival plaque from older adults with or without dementia. Researchers found that the non-dementia samples had a higher proportion of sequences identified belonging to the family *Fusobacteriaceae* and a lower proportion of sequences belonging to *Prevotellaceae* [24]. AD is the most common cause of dementia, and we found a similarly higher proportion of *Fusobacteriaceae* in the controls from this study. In addition, the higher prevalence of genus *Porphyromonas* in normal control group was similar to our finding [13,47], which could be further explored in the future.

Although elderly individuals are more likely to have untreated dental diseases, in this study, patients with AD had the worst overall poor oral health condition. Recently, it has been found that *S. mutans* cell-surface localized adhesin antigen P1 and particular proteins can develop amyloid fibers and cause amyloid formation [21]. Researchers found a higher abundance of *Streptococcus* in the feces of patients with AD compared with normal individuals [22]. These bacteria can lead to dysbiosis of oral microbiota, induce amyloid plaque formation in the brain, and further activate microglia and astrocytes, which are the prominent features of AD [48]. Meanwhile, the significantly higher proportion of Fusobacteriaceae and *Cardiobacterium* in the oral microbiota of the controls could protect them from cognitive decline [24]. Therefore, this platform could serve as a non-invasive diagnostic test for early detection of AD.

There are some limitations that could be improved upon in future studies. First, the PacBio platform required a higher cost than the other tests did. A higher demand could lower the cost and make this test more accessible. Second, the gut microbiota of the participants was not investigated, even though it could be associated with the onset of AD and oral microbiota. The oral dysbiosis could affect the gut microbiota, causing disruption of brain function and pathogenesis of AD via the microbiota–gut–brain axis. A comparison of the compositions of gut and oral microbiota might improve the feasibility of the clinical application of this study in the future. Third, although the correlation between the specific microbiota composition and clinical signs of patients with AD has partially been determined, many factors remain to be studied, such as the influence of partial edentulism, the interaction of other oral microflora, functional level of remaining teeth, presence of fixed or removable prosthesis, and biting force. There is a high likelihood that the disease is multifactorial, and the biomarkers of microbiota composition alone may not sufficiently determine the pathogenesis and diagnosis of AD. Further, although we investigated the differences in the oral microbiota between patients with AD and elderly controls, many confounding factors such as daily habits of the participants, socioeconomic conditions, medications, and comorbidities might have influenced the results. Future studies with a large sample size and longer follow-up are warranted to monitor the outcome of AD.

## 5. Conclusions

To the best of our knowledge, this is the first study to use the PacBio SMRT sequencing platform to determine the connection between oral microbiota and patients with AD. The findings suggest that this platform may offer precise biomarkers for detection of AD. Patients with AD had significantly more missing teeth and higher dental plaque weight, but lower microbial diversity, compared with controls. The proportion of *Lactobacillales*, *Streptococcaceae*, and the *Firmicutes*/*Bacteroidetes* ratios were significantly increased, whereas *Fusobacterium* and Proteobacteria were significantly decreased in patients with AD.

## Figures and Tables

**Table 1:** Characteristics of patients with Alzheimer’s disease (AD) and elderly controls.

Basic Profile	Patients with AD	Elderly Controls
Sample size, *N*	17	18
Male: Female	6: 11	6:12
Age, years	77.9 ± 10.5	65.2 ± 24.6
CDR, score	1.0 (0.5–2.0)	-
CDR-SB, score	7.0 (3.0–12.0)	-
MMSE, score	14 (6.0–21)	-
Decayed, Missing, and Filled Teeth (DMFT) index	17 (12–26)	21 (16–22)
Number of decayed teeth	2 (1–3)	2 (1–3)
Number of missing teeth	10 (5–19) *	5 (3–11)
Number of filled teeth	4 (3–9)	9 (4–12)
Dental plaque weight, g	0.05 * (0.02–0.07)	0.02 (0.02–0.04)

* *p* < 0.05 by two-tailed *t*-test or Mann–Whitney U test, where appropriate. Number of missing teeth and dental plaque weight were significantly larger in the AD group than in the control group (* *p* < 0.05). Clinical Dementia Rating (CDR) score, CDR-SB (CDR–Sum of Boxes), and MMSE (Mini–Mental Status Examination) scores were listed in the AD group.

**Table 2:** Number of unique tags, operational taxonomic units (OTUs), Good’s coverage, and alpha diversity estimates of oral bacterial communities in the AD and control groups.

Group		Patients with AD	Elderly Controls
Unique tags		8659 ± 1792 *	10,486 ± 2637
No. of OTUs		5569 ± 1115 *	6696 ± 1666
Good’s coverage		0.996 ± 0.002	0.997 ± 0.001
Richness index	Chao1	227.94 ± 61.47	236.04 ± 42.65
Diversity index	Shannon	5.38 ± 0.97	5.64 ± 0.51
	Simpson reciprocal	26.59 ± 12.78	29.47 ± 11.56

* *p* < 0.05 by two-tailed *t*-test.

## References

[1] Prince M., Ali G.-C., Guerchet M., Prina A.M., Albanese E., Wu Y.-T. (2016). Recent global trends in the prevalence and incidence of dementia, and survival with dementia. Alzheimer’s Res. Ther..

[2] DeTure M.A., Dickson D.W. (2019). The neuropathological diagnosis of Alzheimer’s disease. Mol. Neurodegener..

[3] Paganini-Hill A., White S.C., Atchison K.A. (2012). Dentition, Dental Health Habits, and Dementia: The Leisure World Cohort Study. J. Am. Geriatr. Soc..

[4] Batty G.-D., Li Q., Huxley R., Zoungas S., Taylor B.-A., Neal B., de Galan B., Woodward M., Harrap S.-B., Colagiuri S. (2013). Oral disease in relation to future risk of dementia and cognitive decline: Prospective cohort study based on the Action in Diabetes and Vascular Disease: Preterax and Diamicron Modified-Release Controlled Evaluation (ADVANCE) trial. Eur. Psychiatry.

[5] Zhuang Z.-Q., Shen L.-L., Li W.-W., Fu X., Zeng F., Gui L., Lü Y., Cai M., Zhu C., Tan Y.-L. (2018). Gut microbiota is altered in patients with Alzheimer’s disease. J. Alzheimer’s Dis..

[6] DeGruttola A.K., Low D., Mizoguchi A., Mizoguchi E. (2016). Current understanding of dysbiosis in disease in human and animal models. Inflamm. Bowel Dis..

[7] Spielman L.J., Gibson D.L., Klegeris A. (2018). Unhealthy gut, unhealthy brain: The role of the intestinal microbiota in neurodegenerative diseases. Neurochem. Int..

[8] Dominy S.S., Lynch C., Ermini F., Benedyk M., Marczyk A., Konradi A., Nguyen M., Haditsch U., Raha D., Griffin C. (2019). Porphyromonas gingivalis in Alzheimer’s disease brains: Evidence for disease causation and treatment with small-molecule inhibitors. Sci. Adv..

[9] Tonomura S., Ihara M., Kawano T., Tanaka T., Okuno Y., Saito S., Friedland R.P., Kuriyama N., Nomura R., Watanabe Y. (2016). Intracerebral hemorrhage and deep microbleeds associated with cnm-positive Streptococcus mutans; a hospital cohort study. Sci. Rep..

[10] Ilievski V., Zuchowska P.K., Green S.J., Toth P.T., Ragozzino M.E., Le K., Aljewari H.W., O’Brien-Simpson N.M., Reynolds E.C., Watanabe K. (2018). Chronic oral application of a periodontal pathogen results in brain inflammation, neurodegeneration and amyloid beta production in wild type mice. PLoS ONE.

[11] Cestari J.A.F., Fabri G.M.C., Kalil J., Nitrini R., Jacob-Filho W., SIQUEIRA J.T.T.d., Siqueira S.R.D. (2016). Oral Infections and Cytokine Levels in Patients with Alzheimer’s Disease and Mild Cognitive Impairment Compared with Controls. J. Alzheimers Dis..

[12] Chen W.-P., Chang S.-H., Tang C.-Y., Liou M.-L., Tsai S.-J.J., Lin Y.-L. (2018). Composition analysis and feature selection of the oral microbiota associated with periodontal disease. Biomed Res. Int..

[13] Yang C.-Y., Yeh Y.-M., Yu H.-Y., Chin C.-Y., Hsu C.-W., Liu H., Huang P.-J., Hu S.-N., Liao C.-T., Chang K.-P. (2018). Oral microbiota community dynamics associated with oral squamous cell carcinoma staging. Front. Microbiol..

[14] Lindahl B.D., Nilsson R.H., Tedersoo L., Abarenkov K., Carlsen T., Kjøller R., Kõljalg U., Pennanen T., Rosendahl S., Stenlid J. (2013). Fungal community analysis by high-throughput sequencing of amplified markers—A user’s guide. New Phytol..

[15] Dassi E., Ballarini A., Covello G., Quattrone A., Jousson O., De Sanctis V., Bertorelli R., Denti M.A., Segata N. (2014). Enhanced microbial diversity in the saliva microbiome induced by short-term probiotic intake revealed by 16S rRNA sequencing on the IonTorrent PGM platform. J. Biotechnol..

[16] Li J., Quinque D., Horz H.-P., Li M., Rzhetskaya M., Raff J.A., Hayes M.G., Stoneking M. (2014). Comparative analysis of the human saliva microbiome from different climate zones: Alaska, Germany, and Africa. BMC Microbiol..

[17] Kumar P.S., Brooker M.R., Dowd S.E., Camerlengo T. (2011). Target region selection is a critical determinant of community fingerprints generated by 16S pyrosequencing. PLoS ONE.

[18] Haffajee A., Socransky S., Patel M., Song X. (2008). Microbial complexes in supragingival plaque. Oral Microbiol. Immunol..

[19] Du Q., Li M., Zhou X., Tian K. (2017). A comprehensive profiling of supragingival bacterial composition in Chinese twin children and their mothers. Antonie Van Leeuwenhoek.

[20] Ardui S., Ameur A., Vermeesch J.R., Hestand M.S. (2018). Single molecule real-time (SMRT) sequencing comes of age: Applications and utilities for medical diagnostics. Nucleic Acids Res..

[21] Oli M., Otoo H., Crowley P., Heim K., Nascimento M., Ramsook C., Lipke P., Brady L. (2012). Functional amyloid formation by Streptococcus mutans. Microbiology.

[22] Li B., He Y., Ma J., Huang P., Du J., Cao L., Wang Y., Xiao Q., Tang H., Chen S. (2019). Mild cognitive impairment has similar alterations as Alzheimer’s disease in gut microbiota. Alzheimer’s Dement..

[23] Poole S., Singhrao S.K., Kesavalu L., Curtis M.A., Crean S. (2013). Determining the presence of periodontopathic virulence factors in short-term postmortem Alzheimer’s disease brain tissue. J. Alzheimer’s Dis..

[24] Cockburn A.F., Dehlin J.M., Ngan T., Crout R., Boskovic G., Denvir J., Primerano D., Plassman B.L., Wu B., Cuff C.F. (2012). High throughput DNA sequencing to detect differences in the subgingival plaque microbiome in elderly subjects with and without dementia. Investig. Genet..

[25] Haririan H., Andrukhov O., Bertl K., Lettner S., Kierstein S., Moritz A., Rausch-Fan X. (2014). Microbial analysis of subgingival plaque samples compared to that of whole saliva in patients with periodontitis. J. Periodontol..

[26] Jack C.R., Bennett D.A., Blennow K., Carrillo M.C., Dunn B., Haeberlein S.B., Holtzman D.M., Jagust W., Jessen F., Karlawish J. (2018). NIA-AA research framework: Toward a biological definition of Alzheimer’s disease. Alzheimer’s Dement..

[27] Haas B.J., Gevers D., Earl A.M., Feldgarden M., Ward D.V., Giannoukos G., Ciulla D., Tabbaa D., Highlander S.K., Sodergren E. (2011). Chimeric 16S rRNA sequence formation and detection in Sanger and 454-pyrosequenced PCR amplicons. Genome Res..

[28] Escapa I.F., Chen T., Huang Y., Gajare P., Dewhirst F.E., Lemon K.P. (2018). New insights into human nostril microbiome from the expanded human oral microbiome database (eHOMD): A resource for the microbiome of the human aerodigestive tract. Msystems.

[29] Segata N., Izard J., Waldron L., Gevers D., Miropolsky L., Garrett W.S., Huttenhower C. (2011). Metagenomic biomarker discovery and explanation. Genome Biol..

[30] Verdam F.J., Fuentes S., de Jonge C., Zoetendal E.G., Erbil R., Greve J.W., Buurman W.A., de Vos W.M., Rensen S.S. (2013). Human intestinal microbiota composition is associated with local and systemic inflammation in obesity. Obesity.

[31] Myer P.R., Kim M., Freetly H.C., Smith T.P. (2016). Evaluation of 16S rRNA amplicon sequencing using two next-generation sequencing technologies for phylogenetic analysis of the rumen bacterial community in steers. J. Microbiol. Methods.

[32] Chen C.-K., Wu Y.-T., Chang Y.-C. (2017). Association between chronic periodontitis and the risk of Alzheimer’s disease: A retrospective, population-based, matched-cohort study. Alzheimer’s Res. Ther..

[33] Goodson J., Groppo D., Halem S., Carpino E. (2009). Is obesity an oral bacterial disease?. J. Dent. Res..

[34] Bajaj J.S., Heuman D.M., Hylemon P.B., Sanyal A.J., White M.B., Monteith P., Noble N.A., Unser A.B., Daita K., Fisher A.R. (2014). Altered profile of human gut microbiome is associated with cirrhosis and its complications. J. Hepatol..

[35] Ling Z., Liu X., Cheng Y., Jiang X., Jiang H., Wang Y., Li L. (2015). Decreased diversity of the oral microbiota of patients with hepatitis B virus-induced chronic liver disease: A pilot project. Sci. Rep..

[36] Shoemark D.K., Allen S.J. (2015). The microbiome and disease: Reviewing the links between the oral microbiome, aging, and Alzheimer’s disease. J. Alzheimer’s Dis..

[37] Durand R., Roufegarinejad A., Chandad F., Rompré P.H., Voyer R., Michalowicz B.S., Emami E. (2019). Dental caries are positively associated with periodontal disease severity. Clin. Oral Investig..

[38] AlQobaly L., Sabbah W. (2020). The association between periodontal disease and root/coronal caries. Int. J. Dent. Hyg..

[39] Sureda A., Daglia M., Castilla S.A., Sanadgol N., Nabavi S.F., Khan H., Belwal T., Jeandet P., Marchese A., Pistollato F. (2020). Oral microbiota and Alzheimer’s disease: Do all roads lead to Rome?. Pharmacol. Res..

[40] Liu Y.C.G., Lan S.-J., Hirano H., Lin L.-M., Hori K., Lin C.-S., Zwetchkenbaum S., Minakuchi S., Teng A.Y.-T. (2020). Update and review of the gerodontology prospective for 2020’s: Linking the interactions of oral (hypo)-functions to health vs. systemic diseases. J. Dent. Sci..

[41] Locker D., Ford J., Leake J. (1996). Incidence of and risk factors for tooth loss in a population of older Canadians. J. Dent. Res..

[42] Marsh P., Martin M. (2009). Oral Microbiology Text and Evolve eBooks Package.

[43] Shimazaki Y., Soh I., Saito T., Yamashita Y., Koga T., Miyazaki H., Takehara T. (2001). Influence of dentition status on physical disability, mental impairment, and mortality in institutionalized elderly people. J. Dent. Res..

[44] Emoto T., Yamashita T., Sasaki N., Hirota Y., Hayashi T., So A., Kasahara K., Yodoi K., Matsumoto T., Mizoguchi T. (2016). Analysis of gut microbiota in coronary artery disease patients: A possible link between gut microbiota and coronary artery disease. J. Atheroscler. Thromb..

[45] Wu Y.-F., Hsu P.-S., Tsai C.-S., Pan P.-C., Chen Y.-L. (2018). Significantly increased low shear rate viscosity, blood elastic modulus, and RBC aggregation in adults following cardiac surgery. Sci. Rep..

[46] Emery D.C., Shoemark D.K., Batstone T.E., Waterfall C.M., Coghill J.A., Cerajewska T.L., Davies M., West N.X., Allen S.J. (2017). 16S rRNA next generation sequencing analysis shows bacteria in Alzheimer’s post-mortem brain. Front. Aging Neurosci..

[47] Yasunaga H., Takeshita T., Shibata Y., Furuta M., Shimazaki Y., Akifusa S., Ninomiya T., Kiyohara Y., Takahashi I., Yamashita Y. (2017). Exploration of bacterial species associated with the salivary microbiome of individuals with a low susceptibility to dental caries. Clin. Oral Investig..

[48] Aguayo S., Schuh C.M.A.P., Vicente B., Aguayo L.G. (2018). Association between Alzheimer’s Disease and Oral and gut microbiota: Are pore forming proteins the missing link?. J. Alzheimer’s Dis..

